# Relationship between resting-state functional connectivity and change in motor function after motor imagery intervention in patients with stroke: a scoping review

**DOI:** 10.1186/s12984-023-01282-w

**Published:** 2023-11-18

**Authors:** Kenya Tanamachi, Wataru Kuwahara, Megumi Okawada, Shun Sasaki, Fuminari Kaneko

**Affiliations:** 1 Department of Physical Therapy, Graduate School of Health Sciences, Tokyo Metropolitan University, 7-2-10 Higashi-Ogu, Arakawa-Ku, Tokyo, Japan; 2 Department of Rehabilitation Medicine, Keio University School of Medicine, 35 Shinanomachi, Shinjuku-ku, Tokyo, Japan

**Keywords:** Motor imagery, Brain–computer interface, Resting state functional connectivity, Stroke, Scoping review

## Abstract

**Background:**

In clinical practice, motor imagery has been proposed as a treatment modality for stroke owing to its feasibility in patients with severe motor impairment. Motor imagery-based interventions can be categorized as open- or closed-loop. Closed-loop intervention is based on voluntary motor imagery and induced peripheral sensory afferent (e.g., Brain Computer Interface (BCI)-based interventions). Meanwhile, open-loop interventions include methods without voluntary motor imagery or sensory afferent. Resting-state functional connectivity (rs-FC) is defined as a significant temporal correlated signal among functionally related brain regions without any stimulus. rs-FC is a powerful tool for exploring the baseline characteristics of brain connectivity. Previous studies reported changes in rs-FC after motor imagery interventions. Systematic reviews also reported the effects of motor imagery-based interventions at the behavioral level. This study aimed to review and describe the relationship between the improvement in motor function and changes in rs-FC after motor imagery in patients with stroke.

**Review process:**

The literature review was based on Arksey and O’Malley’s framework. PubMed, Ovid MEDLINE, Cochrane Central Register of Controlled Trials, and Web of Science were searched up to September 30, 2023. The included studies covered the following topics: illusion without voluntary action, motor imagery, action imitation, and BCI-based interventions. The correlation between rs-FC and motor function before and after the intervention was analyzed. After screening by two independent researchers, 13 studies on BCI-based intervention, motor imagery intervention, and kinesthetic illusion induced by visual stimulation therapy were included.

**Conclusion:**

All studies relating to motor imagery in this review reported improvement in motor function post-intervention. Furthermore, all those studies demonstrated a significant relationship between the change in motor function and rs-FC (e.g., sensorimotor network and parietal cortex).

## Background

Stroke is one of the most prevalent neurological diseases worldwide. Importantly, it induces motor dysfunction and hinders the performance of activities of daily living. Up to 85% of survivors experience hemiparesis immediately after the stroke, resulting in impaired upper extremity function. In addition, 55 to 75% of survivors continue to experience limitations in upper extremity function, even at 3–6 months after the event, further leading to a decline in health-related quality of life [1, 2].

Motor imagery involves as the mental presentation of an action without voluntary body movement [3]. The physiological effect was first represented as the regional cerebral blood flow during rest and planning of voluntary movement reported by Roland et al. [4] They reported that motor imagery activates central sites involved in normal voluntary movements. After more than 10 years, Yahagi and Kasai reported that the primary motor cortex excitability increased during motor imagery without gain modulation in the spinal reflex, using transcranial magnetic stimulation (TMS) [5, 6]. After their reports using TMS, a number of studies concerning the physiological effects of motor imagery have since reported [7–10]. In 2011, Aoyama demonstrated that the facilitatory effect depends on the voluntary effort level of the motor image in the soleus muscle without a change in H-reflex gain [11]. We previously demonstrated the negative effect of sustained rest during joint immobilization on facilitatory function of motor imagery [12]. In that research, we demonstrated that the facilitation effect in which the motor evoked potentials (MEP) during motor imagery was suppressed after immobilization along parallel with decreased muscular output. Furthermore, when the voluntary muscular activity level recovered to that of before the immobilization, MEP was restored to the normal level. These previous findings of the physiological effects indicate the potential use of motor imagery as a clinical treatment, including in stroke. Systematic reviews reported the effects of motor imagery for corticomotoneuronal excitability. In 2019, Dilena et al. [13] indicated that the excitability of the corticomotoneuronal system was enhanced by the kinesthetic illusion, which was induced through visual and tendon vibration.

An advantage of motor imagery intervention is that it may be feasible in patients with severely impaired motor function. Zimmermann-Schlatter et al. [14] conducted a systematic review in 2008 on the efficacy of motor imagery intervention in post-stroke rehabilitation. They found that an improvement in motor imagery, as evidenced by the Fugl-Meyer Assessment (FMA), can confer additional benefits to conventional therapy. Biosignal-based brain-computer interface (BCI) hold great potential for the motor rehabilitation of patients with stroke. Systematic reviews and meta-analyses reported the effects of BCI-based interventions. In 2017, Monge-Pereira et al. [15] indicated that BCI may be potentially beneficial in improving motor outcome measures, such as FMA, Action Research Arm Test (ARAT), and Wolf Motor Function Test (WMFT), in patients with stroke. Carvalho et al. [16] also reported in 2019 that BCI-based intervention, in conjunction with physical practice (conventional or robot-assisted therapy), can enhance upper limb functional recovery. BCI-based intervention can categorize as a closed-loop intervention. In closed-loop intervention, voluntary motor imagery is examined through electroencephalography (EEG). The EEG-based BCI system uses this signal to drive exoskeletal robots and induces peripheral sensory afferent. Meanwhile, open-loop intervention involves methods without voluntary effort to reproduce motor imagery and/ or the absence of sensory afferent. Motor imagery interventions have been shown to be effective in improving motor function [17, 18].

Resting-state functional connectivity (rs-FC) is a powerful tool for exploring the baseline characteristics of brain connectivity. rs-FC is a significantly temporal correlated signal between functionally-related brain regions in the absence of any stimulus or task [19]. rs-FC measurable through functional magnetic resonance imaging (fMRI), EEG, and magnetoencephalography (MEG). According to previous studies, the intensity of rs-FC was related to behavioral measures, and repetition of a specific task modified the rs-FC between brain regions closely related to that task after stroke [20–23].

Previous studies investigating motor imagery intervention reported that the inter-and intra-hemispheric rs-FC differs in patients depending on stroke severity, becoming weak in patients with severe stroke [24]. Patients in the subacute-to-chronic phase after stroke exhibit diminished rs-FC between the primary motor cortex (PMC) of each hemisphere compared to the healthy controls [25]. The index of asymmetry was significantly correlated with motor function deficits, and the rs-FC between postcentral gyrus (S1) and other regions indicated an asymmetrical difference [26], while rs-FC was increased in the ipsilesional (ipsi-) sensorimotor cortex during the neurofeedback intervention [27]. We investigated that relationship between rs-FC and motor function [28]. This study indicated indicate a linear relationship between rs-FC and improvement of motor function. Investigating the relationship between improvements in motor function and changes in rs-FC through motor imagery intervention can provide valuable insights into understanding this relationship. We hypothesized that resting-state brain function coupling underlies the improvement in motor function with motor imagery intervention. However, our hypothesis has not been reviewed. Therefore, the present review aimed to review and describe the status of these studies.

## Methods

### Search strategy

The literature review was based on the framework by Arksey and O’Malley [29]. The following describes the phases of the framework adopted to conduct the scoping study: stage 1: identifying the research question; stage 2: identifying relevant studies; stage 3: study selection; stage 4: charting the data; and stage 5: collating, summarizing, and reporting the results.

The PubMed, Ovid MEDLINE, Cochrane Central Register of Controlled Trials, and Web of Science databases were accessed, and the search was completed on September 30, 2023.

The primary search was conducted using the following terms: ((((illusion) OR ("motor image") OR ("motor imagery") OR ("motor images") OR ("mental practice") OR (Vibrator) OR (Vibration) OR (imitation) OR ("visual stimulation") OR (BCI) OR (BMI) OR ("brain computer interface") OR ("brain machine interface")) AND ((stroke) OR (CVA) OR ("cerebrovascular accident") OR ("cerebral infarct") OR ("cerebral hemorrhage") OR (hemiplegia) OR (hemiparesis) OR (ABI) OR ("acquired brain injury"))) AND ((fMRI) OR (MEG) OR (MRI) OR (TMS) OR ("Transcranial Magnetic Stimulation") OR (PET) OR (EEG) OR ("cortical network")) AND ("resting state")).

Additionally, the following parameters were employed to identify the relevant studies: clinical trials/randomized controlled trials and other studies written in English language whose full texts were available. The publication date ranged from 2011 to 2023. Additional studies were identified via a manual search and duplicates were eliminated.

### Screening

The inclusion criteria were as follows:

- adult stroke patients (age > 18 years).
- treatment using the illusion without voluntary action, motor image, action imitation, and BCI-based interventions.
- the outcome measures included brain function tests, such as fMRI, MEG, transcranial magnetic stimulation, positron emission tomography, and EEG, and motor function tests, such as the FMA, modified Ashworth Scale (MAS), Box and Block Test, and ARAT.
- statistical analysis of the correlation between brain function and motor function before and after the intervention.
- studies published in English language.

The exclusion criteria were as follows:

- interventions such as mirror therapy and brain stimulation.
- non-peer-reviewed studies and papers that reported only the protocol.

Two reviewers screened potential studies to eliminate irrelevant studies based on the reference selection process and above-mentioned inclusion and exclusion criteria. If the relevance of a study was ambiguous from the abstract, it was ascertained from the full text.

### Data extraction and summarization

**Fig. 1:**
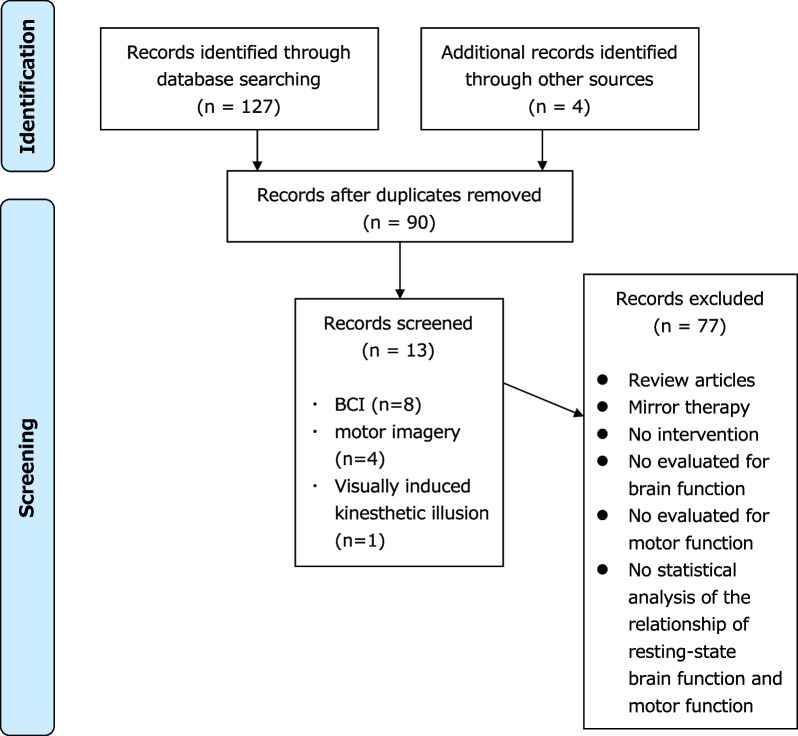
A flow diagram of the selection process

Data on the study design, participants, sample size, interventions used, intervention protocol, evaluation tools, and the outcome of the correlation between brain function and motor function were extracted (Fig. 1). The extracted data were summarized into a table by one independent author.

## Result

### Study selection

The characteristics of the included studies are listed in Tables 1 and 2. A total of 131 studies were identified through the database search, of which 41 were duplicates. Screening by two independent researchers yielded 13 studies on BCI-based interventions [30–37], motor imagery [38–41], and visually induced kinesthetic illusion (V-Kinesthetic illusion) [28] which were included.

**Table 1:** Literature summary of the open-loop intervention

Author (year)	After onset	Sample size	Type of intervention	Task	Number of sessions	CRT	Outcome measure of brain function	Outcome of motor function	Relationship between rs-FC and motor function
Wang X et al. (2020) [38]	121.19 ± 37.33 days	16	Motor imagery training	Imagine basic movements, such as opening and closing of the hand, arm elevation, flexion and extension of the elbow	・ All patients received 3 h of CRT per day, 5 days per week for 4 weeks ・After each day’s CRT, patients received Motor imagery training (30 min)	◯	fMRI	FMA, MBI	・ Fractional amplitude of low-frequency fluctuations in the slow-5 band in the ipsi-IPL was positively correlated with the FMA score ・ The ipsi-IPL with the bilateral PHG was increased after motor imagery intervention correlated with the improvement of FMA score ・ The ipsi-IPL with the bilateral MCG, the cont-MeFG was decreased after motor imagery intervention correlated with the improvement of FMA score
Wang H et al. (2019) [39]	121.19 ± 37.33 days	16	Motor imagery training	Imagine basic movements, such as opening and closing of the hand, arm elevation, flexion and extension of the elbow	・ All patients received 3 h of CRT per day, 5 days per week for 4 weeks ・After each day’s CRT, patients received Motor imagery training (30 min)	◯	fMRI	FMA	・ The increment of clustering coefficient was significantly positively correlated with improvement of upper limb FMA score
Zhang Y et al. (2016) [40]	59.19 ± 15.52 days	17	Motor imagery training	Imagine the actual motions such as grabbing, pushing, fist clenching, writing and finger tapping	・ 30 min, 30 days + CRT	◯	fMRI	FMA	・ Positive correlation between bilateral PMC and the FMA score
Bajaj S et al. (2015) [41]	10.15 ± 13.40 months	13	Motor imagery training	Imagine the actual motions such as 1) brushing or combing hair, 2) picking up and bringing different types of fruit to their mouth, 3) extending their arm to pick up a cup from a cabinet and place it on the counter and gently release it, and 4) cleaning the kitchen counter using a cloth	・ 4 h per day (8-30 min session)	◯	fMRI (Spectral Granger causality measures)	FMA	・ Positive correlation between Granger causality differences (from SMA to lateral PMC) and FMA score in the motor imagery intervention group
Kaneko et al. (2019) [28]	44 ± 29.01 months	11	V-Kinesthetic illusion	Video observation of hand opening and closing	・ 20 min V-Kinesthetic illusion + 60 min CRT, 10 days	◯	fMRI	FMA, MAS, ARAT, MAL	・ Changes in the relationship (negative to positive) between motor function and the interhemispheric rs-FC of the bilateral IPS, and disappearance of the significant negative correlation between the motor functions and the interhemispheric rs-FC of the IPL in the unaffected side and PMC occurred in parallel with the behavioral changes

*ANG* angular gyrus, *ARAT* Action Reach Arm Test, *CRT* conventional rehabilitation, *FMA* Fugle-Meyer Assessment, *fMRI* functional magnetic resonance imaging, *IFG* inferior frontal gyrus, *INS* insula, *IPL* inferior parietal lobule, *IPS* intraparietal sulcus, *IOG* inferior occipital gyrus, *MAL* Motor activity Log, *MAS* modified Ashworth Scale, *MBI* modified Barthel index, *MCG* middle cingulate gyrus, *MEG* magnetoencephalography, *MeFG* medial frontal gyrus, *MiFG* middle frontal gyrus, *PHG* parahippocampal gyrus, *PMC* premotor cortex, *rs-FC* resting state functional connectivity, *SFG* superior frontal gyrus, *SMA* supplementary motor area, *STG* superior temporal gyrus, *ipsi-* ipsilesional, *cont-* contralesional

**Table 2:** Literature summary of the closed-loop intervention

Author (year)	After onset	Sample size	Type of intervention	Task	Number of sessions	CRT	Outcome measure of brain function	Outcome of motor function	Relationship between rs-FC and motor function
Rustamov N et al. (2023) [30]	68.2 ± 78.2 months	30	EEG-based BCI	Imagine opening and closing their affected hand	60 sessions (5sessions/week) 60 min	×	EEG	FMA, ARAT, Motricity Index, Gross Grasp, MAS	・ FMA, ARAT and Motricity Index significant correlated with an increased coupling of theta and gamma frequencies in the motor regions
Ma ZZ et al. (2023) [31]	5.90 ± 2.99 months	20	EEG-based BCI	Imagine the state of the muscles as the right or left-hand stretches or contracts	10 sessions (5sessions/week) 40 min	×	EEG, fMRI	FMA	・ The assortativity of the dorsal attention network was positively correlated with the gain of the FMA-UE after treatment
Rustamov N et al. (2022) [32]	65.7 ± 15.5 months	17	EEG-based BCI	Imagine opening and closing their affected hand	60 sessions (5sessions/week) 60 min	◯	EEG	FMA, AMAT, Motricity Index, MAS	・ Theta–gamma coupling in bi-PMC was enhanced and was significantly correlated across BCI intervention sessions
Yuan K et al. (2021) [33]	3.98 ± 3.05 years	14	EEG-based BCI	Imagine either grasping or releasing a cup following the instruction displayed on the monitor	20 sessions (3–5 sessions/week) 30 min	×	EEG + fMRI	FMA	・ Information flow change from cont-BA6 to ispsi-PMC and SMA to ipsi-PMC significantly correlated with the FMA score change ・ rs-FC change between ipsi-PMC and cont-BA6 was positively correlated with the FMA
Yuan K et al. (2020) [34]	3.98 ± 3.05 years	14	EEG-based BCI	Imagine either grasping or releasing a cup following the instruction displayed on the monitor	20 session (3–5 sessions/week) 30 min	×	fMRI	FMA	・ Correlation analysis also showed that interhemispheric rs-FC change between Pre and Post sessions was significantly correlated with FMA score change ・ FMA score change was significantly correlated with rs-FC change between ipsi-PMC seed and the significant cluster in the cont-premotor area ・ FMA score change was significantly correlated with rs-FC change between ipsi-SMA seed and the significant cluster in bilateral SPL
Wu Q et al. (2019) [35]	2.11 ± 0.3 months	25	EEG-based BCI	Imagine grasping or releasing a cup with the affected hand, after an image-inverted video taken prior of the unaffected hand	20 session (5 sessions/week) 60 min	◯	fMRI	FMA, ARAT, WMFT	・ After comprehensive rehabilitation, including BCI training, increases in rs-FC between the left BA5 and right BA48 were positively correlated with clinical scores post training: upper limb FMA post score, ARAT post score, and WMFT post score
Rathee D et al. (2019) [36]	21.8 ± 1.1 months	4	EEG-based BCI	Presentation of a cue to perform either a left or right-hand grasp attempt	12 sessions 30 min	×	MEG	ARAT, grip- strength	・ The motor network involving the precentral gyrus (i.e., PMC), postcentral gyrus (i.e., S1), and SMA brain regions became stronger with upper limb functional recovery
Várkuti B et al. (2013) [37]	11.67 ± 13.51 months	6	EEG-based BCI	8-Direction reaching with MANUS robot	12 rehabilitation sessions in approximately 1 month	×	fMRI	FMA	・ Increases in rs-FC of the SMA, bi-PMC, and parts of the visuospatial system with mostly association cortex regions and the cerebellum correlated with upper limb functional improvement

*AMAT* Arm Motor Ability Test, *ARAT* Action Reach Arm Test, *BA* Brodmann area, *BCI* brain–computer interface, *CRT* conventional rehabilitation, *EEG* electroencephalography, *FMA* Fugle-Meyer Assessment, *fMRI* functional magnetic resonance imaging, *IPL* inferior parietal lobule, *IPS* intraparietal sulcus, *MAS* modified Ashworth Scale, *MBI* modified Barthel index, *MCG* magnetoencephalography, *MEG* magnetoencephalography, *PHG* parahippocampal gyrus, *PMC* premotor cortex, *rs-FC* resting state functional connectivity, *SMA* supplementary motor area, *SPL* Superior parietal lobule, *WMFT* Wolf Motor Function Test, *ipsi-* ipsilesional, *cont-* contralesional, *bi-* bilateral

### Participants

A total of 73 and 130 participants underwent open- and closed-loop interventions, respectively. The mean interval between the onset of stroke and open-loop intervention was 1.94 to 44.0 months and that for closed-loop intervention was 2.11 to 47.75 months. Three studies that used open-loop intervention evaluated patients in the acute stage (1.94–3.84 months) [38–40] and two studies evaluated patients in the chronic stage (10.15–44.0 months) [28, 41], while two studies that used closed-loop intervention evaluated patients in the acute stage (2.11–5.90 months) [31, 35], and six studies evaluated patients in the chronic stage (11.67–47.75 months). [30, 32–34, 36, 37].

### Training protocol

Open-loop intervention training sessions (e.g., motor imagery therapy) lasted 20–60 min, and the treatment course was 10–30 days. All participants received standard medical care and rehabilitation (conventional therapy). The protocol of the motor imagery task (without voluntary movement) was as follows: (1) opening and closing of the hand [38, 39], (2) arm elevation [38, 39], (3) flexion and extension of the elbow [38, 39], (4) grabbing [40], (5) pushing [40], (6) first clenching [40], (7) writing [40], (8) finger tapping [40], (9) brushing or combing hair [41], (10) picking up and bringing different types of fruit to the mouth [41], (11) extending the arm to pick up a cup from a cabinet, placing it on the counter and gently releasing it [41], (12) cleaning the kitchen counter using a cloth [41], and (13) observing a video of opening and closure of a hand [28].

Closed-loop intervention training was conducted over 10–60 sessions, and the duration of each session was different. Three studies used conventional therapy. The protocol of the motor imagery task was as follows: (1) opening and closing the affected hand [30, 32], (2) the state of the muscles as the hand stretches or contracts [31], (3) grasping or releasing a cup according to the instruction [33–36], and (2) reaching in eight directions [37]. The closed-loop intervention was assisted by a robotic equipment as follows; hand grasp and open [30–36] and the MANUS robot in moving the stroke-affected limb toward the goal displayed on the screen [37].

### Outcome measures

Nine studies used resting-state brain function as evaluated with fMRI as the outcome measure [28, 31, 33–35, 37–41] (one study additionally used EEG [33]), EEG used in two studies, [30, 32] and MEG was used in one study [36].

Motor function outcome measures were used to assess the functional aspects or activities of daily living. Twelve studies used the FMA [28, 30–35, 37–41], five studies used the ARAT [28, 30, 32, 35, 36], three studies used the grip strength [30, 32, 36], two studies used the modified Ashworth Scale (MAS) and Morticity Index [30, 32], and one study used the WMFT [35] to measure upper limb motor function. One study used the modified Barthel index [38] and one study used motor activity log [28] as measures of activities of daily living.

### Relationship between brain function and motor function

Open-loop intervention studies used seed-based FC analysis, graph theory, and spectral Granger causality (GC) measures for MRI analysis. Seed-based FC analysis was used to identify temporally correlated brain regions using blood-oxygen-level-dependent (BOLD) signal fluctuations in the regions of interest. Three studies used seed-based FC analysis, which indicated the following: (1) fractional amplitude of low-frequency fluctuations in the slow-5 band in the ipsi-inferior parietal gyrus (IPL) was positively correlated with the FMA [38]; (2) The ipsi-IPL with the bilateral (bi-) parahippocampal gyrus (PHG) was increased after motor imagery intervention correlated with the improvement of FMA score [38]; (3) The ipsi-IPL with the bi-middle cingulate gyrus (MCG), the contralesional (cont-) medial frontal gyrus (MeFG) was decreased after motor imagery intervention correlated with the improvement of FMA score. [38]; (4) the rs-FC between the bi-PMC was positively correlated with the FMA [40]; and (5) the relationship (negative to positive) between the interhemispheric rs-FC of the bi-IPS and FMA changes and the significant negative correlation between the FMA and interhemispheric rs-FC of the IPL disappeared on the unaffected side and premotor cortex after the intervention [28]. One study, which used graph theory, indicated that the clustering coefficient was significantly positively correlation with the FMA [26]. The study that used spectral GC measures indicated a positive correlation between GC differences (from the supplementary motor area (SMA) to the lateral premotor cortex) and FMA [41].

Closed-loop intervention studies used seed-based FC analysis and EEG and MEG parameters. Five studies used seed-based FC analysis and reported the following: (1) the rs-FC change between the ipsi-PMC and cont-Brodmann area (BA)6 was positively correlated with FMA [33]; (2) the FMA score altered with changes in the interhemispheric rs-FC [34]; (3) the rs-FC between the ipsi-PMC with the cont-premotor area and ipsi-SMA of the bi-superior parietal lobule (SPL) was positively correlated with FMA [34]; (4) the changes in interhemispheric rs-FC and FMA score were positive correlated [34]; (5) the left BA5 and right BA48 were positively correlated with the FMA, ARAT, and WMFT [35]; (6) the rs-FC of the SMA, cont- and ipsi-PMC, visuospatial system and cerebellum correlated with FMA [35]. Three study used EEG parameter analysis and reported the following: (1) FMA, ARAT and Motricity Index significant correlated with increased coupling of theta and gamma frequencies in the motor regions [30], (2) Theta-gamma coupling was enhanced bi-PMC and showed significant correlations across BCI intervention sessions [32], and (3) the information flow change from the cont-PMA to the ipsi-PMC and SMA to the ipsi-PMC were significantly correlated with the FMA score [33]. One study, which used MEG parameters, reported that the motor network involving the PMC, S1, and SMA brain regions became stronger with upper-limb functional recovery [36].

## Discussion

### Summary of evidence

**Fig. 2:**
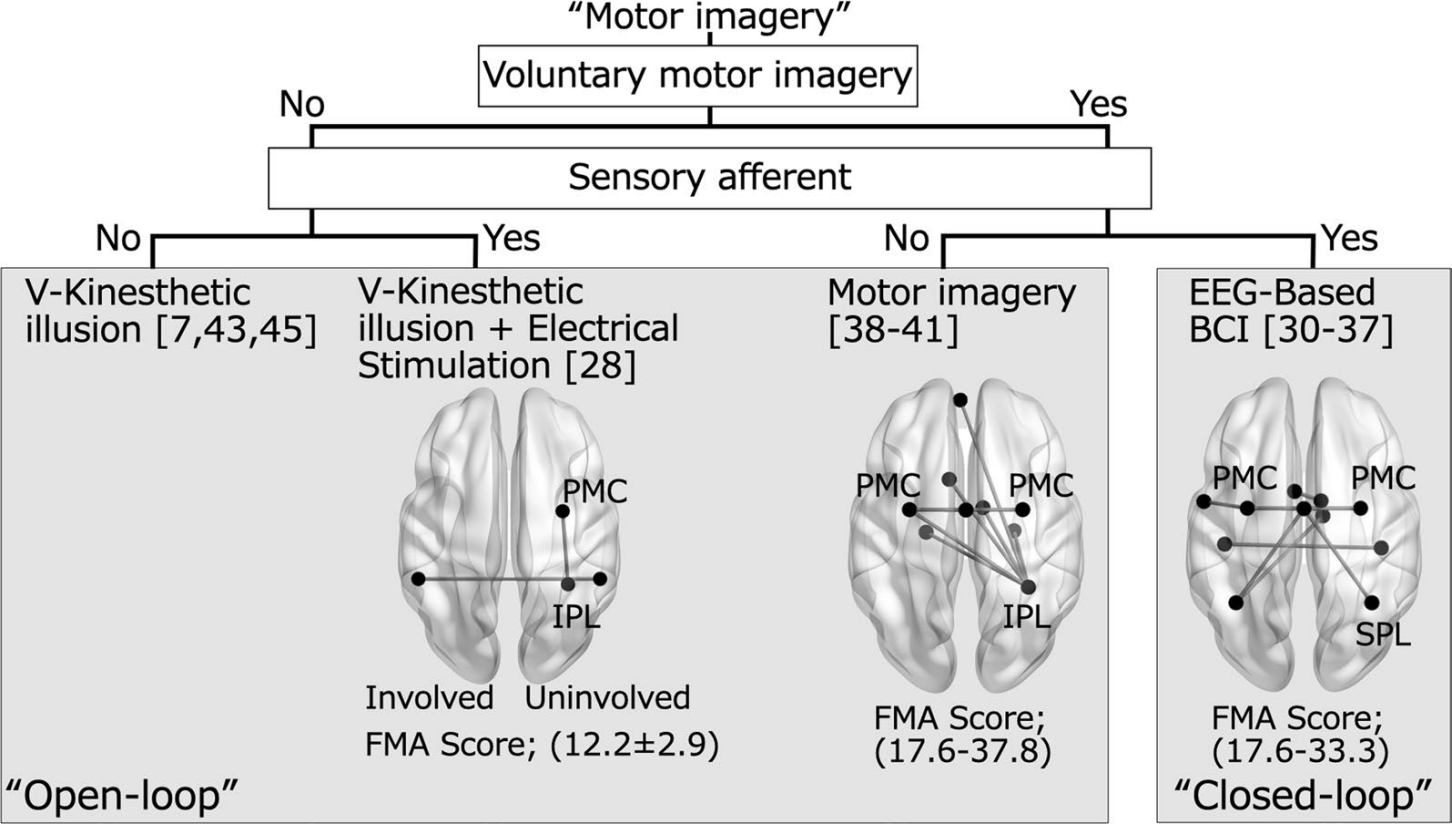
Categories of motor imagery intervention and brain regions associated with improved motor function and changes in rs-FC. The unified right hemisphere as an uninvolved hemisphere. FMA Score indicate range of mean score in before intervention (The article using V-Kinesthetic illusion + Electrical stimulation was indicated as mean ± SD)

The categories of motor imagery interventions extracted in this review and the brain networks that were associated with changes in motor function are summarized in Fig. 2. In closed-loop intervention, voluntary motor imagery is examined through electroencephalography (EEG). The EEG-based BCI system uses this signal to drive exoskeletal robots and induces peripheral sensory afferent. Then, in BCI-based intervention voluntary motor imagery can be modulated based on sensory afferent, we categorized this intervention as a closed-loop. Meanwhile, open-loop intervention involves methods without voluntary effort to reproduce motor imagery and/ or the absence of sensory afferent. All 13 studies were included in this review disturbed motor function after stroke improved following intervention in all studies. Furthermore, the relationship between the change in motor function and rs-FC, including the sensorimotor network and parietal cortex. Previous studies suggested the effectiveness of open- and closed-loop interventions in improving motor function [14–16]. Exploring the correlation between the improvement in motor function and changes in rs-FC following motor imagery intervention can help elucidates the underlying causality behind this relationship.

### Phases of stroke during the intervention

Functional recovery after stroke is known to reach a plateau within 3–6 months, and 85% of stroke survivors experience paralysis and 55–75% have upper limb disfunction, which are associated with a diminished health-related quality of life [1]. All studies selected for this review included patients in the subacute to chronic phase and exhibited recovery of motor function following open- or closed-loop intervention. Both types of intervention have the potential to improve motor function in the chronic phase after stroke.

### Differences between open-loop and closed-loop intervention protocols

The most salient difference between the open-loop and closed-loop intervention protocols was the inclusion of combined conventional therapy in the former. Open-loop intervention was combined with conventional therapy in all studies. Open-loop interventions included motor imagery and V-Kinesthetic illusion. Open-loop intervention entails repetitive cognitive recall of body movements without voluntary body movements as sensory afferent of the motor imagery. V-Kinesthetic illusion is defined as the psychological phenomenon in which a person who is resting feels as if a part of their own body is moving or feels the desire to move a body part while watching film footage of a moving body part [7]. Kaneko et al. refer to this therapy as 'KINVIS therapy' because abbreviated from kinesthetic illusion induced by visual stimulation, and the effect of KINVIS therapy was explored in the clinical trials [10, 28, 42–44]. In several studies, V-Kinesthetic illusion while neuromuscular electrical stimulation was applied to the agonist muscle to the one causing the movement in the film. In V-Kinesthetic illusion, motor imagery was not a picture but kinesthetic perception. In addition to this step, motor imagery is passively induced, and brain activity similar to that during exercise is obtained [45]. Such as V-Kinesthetic illusion, passively induced motor imagery following the cognitive replacement was categorized as open-loop intervention in this review.

Unintentionally generated motor imagery (e.g., observation of movement) is called implicit motor imagery [46]. According to Hanakawa et al. [47, 48], there is additional evidence suggesting that the participants’ neural activity during motor imagery resembled that observed just prior to actual finger movements. V-Kinesthetic illusion is without intentionally movement and passively induced motor imagery during observation movements. V-Kinesthetic illusion can be represented as virtual kinesthetic perception, and it can have markedly vivid kinesthetic perception than simple observation of movement [7]. Therefore, we interpret it as implicit motor imagery.

Closed-loop intervention, which uses BCI as feedback for motor imagery, can activate the neural pathway of motor control via motor intention and the actual movement [49]. BCI-based interventions can ensure better active engagement and motivation of patients compared to conventional therapy [50]. Closed-loop intervention does not necessarily require conventional therapy; EEG-based BCI occur with joint movements associated with motor imagery, which may be equivalent to active movement. [51] In this case, conventional therapy may not be required for impairment of motor function.

### Relationship between brain and motor function after motor imagery intervention

Previous studies used fMRI to show that the motor imagery and perception of a kinesthetic illusion were associated with activation of the primary motor cortex, premotor cortex, SPL, primary somato-sensory cortex, SMA, IPL, cingulate motor area, and cerebellum [52–58]. BCI-based intervention was associated with rs-FC networks related to the motor attempt [59–61].

In 2019, Kaneko et al. [28]. reported that the relationship between the pre-test rs-FC and the improvement in motor function after V-kinesthetic illusion therapy showed a significant negative correlation with affected IPS-unaffected IPS to the ARAT and a positive correlation from affected SMG-Vermis to the ARAT. Miyawaki et al. [62] explored the effect of V-kinesthetic illusion therapy with therapeutic exercise (TheEx) on motor functions through spasticity. They used a mediation model in which the indirect effect was evaluated with path analyses in structural equation modeling. V-Kinesthetic illusion therapy combined with TheEx leads to a reduction in the MAS score, resulting in improvements in FMA and ARAT scores. Notably, the mediation model revealed that there was no significant direct impact of V-Kinesthetic illusion therapy on FMA and ARAT scores. These findings imply that the influence of V-Kinesthetic illusion therapy with TheEx on upper limb motor function is indirectly mediated through its effect on spasticity. Moreover, rs-FC between affected IPS-unaffected IPS was associated with the motor function of hands and fingers, unaffected IPL-unaffected PMd was associated with the motor function of shoulders and elbows and affected IPS-unaffected IPS or affected SMG-Vermis reflect the change in motor function. Thus, investigating the relationship between rs-FC and improvement in motor function with therapy may reveal a causal relationship between the improvement in motor function and therapy.

All studies in this review performed open-loop intervention without voluntary movement and the absence of sensory afferent or closed-loop intervention with sensory afferent of motor imagery using BCI. In both types of interventions, improvements in motor function were related to the change of rs-FC, including the sensorimotor network and parietal cortex. In the closed-loop intervention, proprioceptive and visual inputs were associated with movement. This may induce a sense of body ownership and kinesthetic perception and increase the rs-FC of the motor-related function and parietal cortex.

Previous studies have not sufficiently focus on established treatments to improve severe impairment of upper limb motor function. In our previous clinical trial with V-Kinesthetic illusion [28], the baseline FMA of stroke patients included lower than that of other motor imagery interventions. The V-Kinesthetic illusion is not limited by motor impairment and may be better suited for patients with severe motor paralysis.

Closed-loop interventions (EEG-based BCI) have recently been proposed as a stroke neurorehabilitation strategy to improve symptoms, including paralysis, cognitive disorders, and aphasia [35]. Despite the substantial heterogeneity in the available literature, there is a consensus that closed-loop intervention can help improve upper limb motor function in patients with stroke [16, 63]. Furthermore, the result of this review indicated that closed-loop intervention, as with open-loop, improves motor function and induces the changes in the rs-FC.

Several studies already showed that motor imagery increases corticospinal excitability [5–13]. The changes in rs-FC following the motor imagery intervention in the present study may represent a brain network enhanced by use-dependent plasticity due to repetition of motor imagery [64, 65].

Future studies should examine not only the assessment of motor function but also brain function because the latter may help clarify the mechanism of motor function improvement.

### Limitations

The quality of evidence was not assessed in this review. The type and severity of the participants’ disability, as well as the intervention methodologies and protocols, were not considered. A high-quality systematic review with a larger scope and with control for factors affecting the effectiveness of rehabilitation (e.g., type of lesion, the phase of recovery, dosage, and intensity of training) is necessary to address these limitations in the future.

## Conclusion

This review shows that rs-FC is related with change in motor function after motor imagery intervention. There are many similarities between the open-loop and closed-loop interventions with respect to the brain regions, such as the sensorimotor network and the parietal cortex, which are correlated with the change in motor function. These findings may provide a neurological basis for a clinician considering a motor imagery intervention. It should be noted, however, that the validation of the effectiveness of motor imagery interventions still involves heterogeneity to date. Further studies are required to strengthen the evidence on intervention protocols and provide detailed information regarding the application of different interventions to optimize practice benefit and outcomes.

## Data Availability

Not applicable.

## Abbreviations

AG: Angular gyrus
ARAT: Action Reach Arm Test
BA: Brodmann area
BBS: Berg Balance Scale
BOLD: Blood-oxygen-level-dependent
BCI: Brain–computer interface
EEG: Electroencephalography
EMG: Electromyography
FMA: Fugle-Meyer Assessment
fMRI: Functional magnetic resonance imaging
IFG: Inferior frontal gyrus
INS: Insula
IOG: Inferior occipital gyrus
IPL: Inferior parietal lobule
IPS: Intraparietal sulcus
MAS: Modified Ashworth Scale
MCG: Middle cingulate gyrus
MeFG: Medial frontal gyrus
MEG: Magnetoencephalography
MEP: Motor evoked potential
MiFG: Middle frontal gyrus
MTG: Middle temporal gyrus
PHG: Parahippocampal gyrus
PMC: Primary motor cortex
rs-FC: Resting-state functional connectivity
SFG: Superior frontal gyrus
SMA: Supplementary motor area
SMG: Supramarginal gyrus
SPL: Superior parietal lobule
STG: Superior temporal gyrus
WMFT: Wolf Motor Function Test
